# Reducing the impact of peer victimization on adolescent insomnia: Which is more important, peers or family support?

**DOI:** 10.3389/fpsyt.2025.1487715

**Published:** 2025-02-19

**Authors:** Siyi Hu, Yuxuan Wang, Qirong Wan, Zhenhua Chen, Jingyi Fan

**Affiliations:** ^1^ Developmental and Educational Psychology Institute, Wuhan University, Wuhan, China; ^2^ Mental Health Center, Renmin Hospital of Wuhan University, Wuhan, China; ^3^ Pediatric Department, Zhongnan Hospital of Wuhan University, Wuhan, China

**Keywords:** adolescents, peer victimization, insomnia, friendship quality, family functioning

## Abstract

**Background:**

Adolescent insomnia is a global public health issue, with peer victimization identified as one of the risk factors. However, some adolescents are able to resist the negative impact of peer victimization, and the protective mechanisms underlying this resilience require further clarification. This study aims to evaluate the moderating roles of friendship quality and family functioning on the effects of peer victimization on insomnia.

**Methods:**

Participants (n=506, age=14.49 ± 0.68, 54.5%female) from Hubei, China, completed the Insomnia Severity Index (ISI), Multidimensional Peer Victimization Scale (MPVS), Friendship Quality Questionnaire (FQQ), and Family Adaptation, Partnership, Growth, Affection, and Resolve (APGAR).

**Results:**

The more severe the peer victimization, the higher the level of insomnia among adolescents. Family functioning moderated the relationship between peer victimization and insomnia severity, but no moderating effect of friendship quality was found.

**Conclusion:**

The results suggest that high family functioning can mitigate the impact of peer victimization on insomnia. In the screening and intervention for peer victimization and insomnia among adolescents, the importance of family functioning should be emphasized, while recognizing that friendship quality may not play a significant role.

## 1 Introduction

High-quality sleep is crucial for the physical and mental development of adolescents. The American Academy of Sleep Medicine recommends that adolescents aged 13-18 maintain 8-10 hours of regular sleep each night (1). However, in reality, an increasing number of adolescents report sleep problems. A study across 20 countries showed that in the past century, minors have lost more than one hour of sleep per night (2), with older students being more prone to sleep deprivation (3). Insomnia is a significant manifestation of sleep problems, and the DSM-5 defines insomnia as having difficulty sleeping and impaired daytime functioning for at least three months and occurring at least three times a week (4). In China, the overall prevalence of insomnia symptoms among adolescents is 32% (5), with 28.9% of adolescents experiencing insufficient and inefficient sleep or daytime functional impairment (6). Research has shown a significant relationship between adolescent insomnia, short sleep duration, and weight gain (7). Maintaining sleep difficulties and disorders of arousal are associated with mental health issues (8), and adolescents with short and poor-quality sleep are more likely to exhibit higher levels of internalizing problems, aggression, and rule-breaking behavior (9). Moreover, insomnia can negatively impact attention, learning motivation, and interest (10), and it is a significant negative predictor of academic performance, particularly in mathematics (11).

Multiple factors contribute to sleep problems. Internal factors include changes in sleep rhythms during puberty, while external factors encompass class schedules, use of electronic media, school pressure, and caffeine consumption (12). Additionally, adversity can increase the risk of insomnia (13). During adolescence, issues with peer relationships can be particularly distressing, especially peer victimization, which is defined as repeated attacks, exclusion, and humiliation of the victim. Peer victimization includes relational victimization and physical victimization, with 26.25% to 34.13% of elementary school students in China experiencing frequent peer victimization (14). Different forms of peer victimization can significantly predict adolescents’ internalizing or externalizing problems (15) and are significantly associated with sleep disorders (16), This relationship is often bidirectional: peer victimization can increase pre-sleep worry and fear, while insomnia can affect emotional regulation and coping strategies, thus increasing the risk of peer victimization (17).

A longitudinal study on adolescents found that positive peer relationships significantly predict fewer sleep problems a year later (18). While the peer support quantity and quality have different effects on adolescents, longitudinal results showed that the quality of peer support reported during adolescence is a better predictor of depressive symptoms than the quantity, and depression is associated with sleep problems (19). Friendship quality is a measure of the intimacy in peer relationships. Elementary school students with high friendship quality reported better life satisfaction and fewer mental health issues (20), though there is less research on the relationship between friendship quality and adolescent insomnia. Studies have found a significant bidirectional association between interpersonal adjustment and sleep quality in college students, with higher friendship quality predicting better sleep quality (21). Additionally, the quality of friendships during adolescence can play a protective role between peer victimization and mental health. However, other studies have found no such association or have found that high friendship quality can exacerbate the distress associated with peer victimization (22).

Family, as another fundamental type of social support, is closely related to psychological health (23). It refers to the emotional communication and problem-solving abilities among family members. Adolescents spend more time at home than at school, and family functioning significantly impacts their sleep. For instance, a study on 3,636 preschool children in rural China found that lower family functioning is associated with a higher risk of sleep disorders (24). Meta-analyses indicated that parents can enhance adolescents’ sleep. Healthy family functioning and parental warmth can improve sleep quality and daytime functioning in adolescents (25). Longitudinal study found similar results, showing that greater child-reported parental warmth is associated with longer sleep duration, greater morningness preference, and less daytime sleepiness (26). Although many studies have explored the direct relationship between family functioning and sleep issues across various populations, there is limited research specifically focused on adolescents.

This study controls for the impact of demographic variables on the moderating effect and investigates the relationship between peer victimization and insomnia in a sample of Chinese adolescents. It also examines whether friendship quality and family functioning can mitigate or exacerbate the impact of peer victimization on insomnia. The aim is to help schools and families understand and identify the challenges adolescents may face, promoting the healthy development of vulnerable adolescents both physically and mentally. In this study, we propose the following hypotheses: (1) Peer victimization positively predicts the level of insomnia; (2) Friendship quality has a negative moderating effect on the relationship between peer victimization and insomnia; (3) Family functioning has a negative moderating effect on the relationship between peer victimization and insomnia.

## 2 Methods

### 2.1 Participants and procedure

This study employed cluster sampling to select adolescents about to enter the second or third year of junior high school from a public school in Hubei Province, China. A total of 531 questionnaires were collected, with 25 excluded due to more than 10% of items being unanswered, resulting in 506 valid responses. The demographic characteristics of the participants are shown in **Table 1**. There were 230 males (45.5%) and 276 females (54.5%), with ages ranging from 12 to 15 years (*M*= 13.49, *SD*=0.68). Most participants had rural household registration (n=352, 69.57%), and their parents’ education levels were predominantly junior high school graduation—fathers: 273 (53.95%), mothers: 282 (55.73%). Family economic conditions were generally moderate (n=277, 53.74%). Specifically, the per capita disposable income of wealthy family economic status is 90,116 yuan, the good family economic status is 47,397 yuan, the average family economic status is 30,598 yuan, and the poor family economic status is 8,601 yuan (27). Nearly half of the families had fathers and/or mothers working far away from home (n=241, 47.63%), with 17.98% (n=91) being away for more than a year. Some participants’ parents were currently separated, divorced, or deceased (n=134, 26.48%).

**Table 1:** Summary of the participants’ demographic characteristics (N=506).

Variable	Cases (*n*)	Proportion (*%*)
Gender		
Male	230	45.45
Female	276	54.55
Grade		
Junior high school year one	233	56.05
Junior high school year two	273	53.95
Only child		
Yes	264	52.17
No	242	47.83
Household Registration		
Urban	154	30.43
Rural	352	69.57
Parent Working Away		
No	265	52.37
Father	171	33.79
Mother	21	4.15
Both parents	49	9.68
Father’s Education		
Elementary or below	83	16.40
Junior High School	273	53.95
Senior High School	132	26.09
College or above	18	3.56
Mather’s Education		
Elementary or below	68	13.44
Junior High School	282	55.73
Senior High School	141	27.87
College or above	15	2.96
Parents’ Marital Status		
Living Together	372	73.52
Separated	43	8.50
Divorced	83	16.40
One or both deceased	8	1.58
Family Economic Status		
Poor	23	4.55
Average	277	53.74
Good	195	38.54
Wealthy	11	2.17

After obtaining approval from the school principal, the participants’ legal guardians/next of kin signed informed consent forms to participate in this study. In June 2023, the researchers conducted a questionnaire survey among the participants in various classrooms, with the completed questionnaires collected on-site. The administration process took approximately 30 minutes. Participants took approximately 10-15 minutes to complete the questionnaires for this study, with additional time allocated for completing other surveys.

### 2.2 Measures

#### 2.2.1 Insomnia Severity Index (ISI)

The Chinese version of the ISI (28) consists of 7 items, designed to assess an individual’s self-perceived insomnia and its impact in the past month. The items are rated on a 5-point Likert scale, with total scores ranging from 0-7 indicating absence of insomnia, 8-14 indicating sub-threshold insomnia, 15-21 indicating moderate insomnia, and 22-28 indicating severe insomnia. The Cronbach’s α of this scale in this study was.71.

#### 2.2.2 Multidimensional Peer Victimization Scale (MPVS)

Peer victimization issues were analyzed using the Chinese version of the MPVS (29). The MPVS includes two dimensions of physical and relational victimization, with a total of 11 items, using a 4-point scoring system. Adolescents with a score of 0 on physical or relational victimization factors are not victimized, 0 to 2 are sometimes victimized, and 2 to 3 are frequently victimized. The higher the score, the deeper the degree of peer victimization. The Cronbach’s α of this scale in this study was.91.

#### 2.2.3 Friendship Quality Questionnaire (FQQ)

The Chinese version of the FQQ was adopted (30). The questionnaire consists of 18 items rated on a 5-point scale and includes six dimensions: intimacy exchange, validation and caring, help and guidance, conflict resolution strategies, companionship and recreation, and conflict and betrayal. Participants were asked to answer based on the actual situation between them and their best friend. The first five dimensions represent positive characteristics of a best friendship, while the last dimension represents negative qualitative aspect of the best friendship. A higher total score indicates better friendship quality. The Cronbach’s α of this scale in this study was.91.

#### 2.2.4 Family APGAR (Adaptation, Partnership, Growth, Affection, and Resolve)

Family functioning was measured using the Chinese version of the Family APGAR (31). The scale includes 5 items that assess family adaptation, partnership, growth, affection, and resolve. Each item is scored from 0 to 2, representing “seldom happens,” “sometimes happens,” and “often happens,” respectively. A total score of 0-3 indicates severe family dysfunction, 4-6 indicates moderate family dysfunction, and 7-10 indicates good family functioning. The Cronbach’s α of this scale in this study was.94.

### 2.3 Data analysis

This study used SPSS 26.0 software for data organization and analysis. Independent samples t-tests and one-way ANOVA were applied for group comparisons. Pearson correlation analysis was used to explore relationships, while hierarchical regression was conducted to perform regression analysis and test moderating effects.

## 3 Results

### 3.1 Descriptive analysis

Descriptive analysis results (see **Table 2**) indicated that 40.32% of adolescents experienced insomnia, 57.51% had less than 7 hours of sleep per night, and people with insomnia were included. Insomnia issues were primarily related to difficulty falling asleep (57.71%), difficulty maintaining sleep (40.51%), early awakening (40.51%), and discomfort such as nightmares (32.21%). Additionally, 69.96% reported that sleep problems interfered with daytime functioning, and 56.52% reported that sleep problems affected their quality of life. Among the participants, 2.96% were currently taking sleeping medication.

**Table 2:** Distribution of the research variables (N=506).

Variable	Cases (*n*)	Proportion (*%*)
Family Functioning (APGAR)		
Severe Dysfunction	162	32.02
Moderate Dysfunction	177	34.98
Good Function	167	33.00
Insomnia Severity (ISI)		
absence of insomnia	302	59.68
sub-threshold insomnia	159	31.42
moderate insomnia	40	7.91
severe insomnia	5	0.99

64.03% of adolescents have experienced peer victimization, among which 55.34% reported relationship victimization and 41.90% reported physical victimization. The mean score for relational victimization (0.43 ± 0.60) was higher than for physical victimization (0.37 ± 0.59). The average score for friendship quality was 3.46 ± 0.65, with high-quality friendships primarily reflected in companionship and recreation (3.97 ± 1.03), absence of conflict and betrayal (3.87 ± 1.00), and conflict resolution strategies (3.80 ± 1.07). The average score of family functioning was 1.04 ± 0.68, the overall level was low, only 33% of adolescents reported good.

### 3.2 Differences in demographic variables

According to **Table 3**, there were significant differences in friendship quality (*t*=-2.83, *p*<.01), family functioning (*t*=2.57, *p*<.05), and insomnia severity (*t*=-2.68, *p*<.01) based on gender. Insomnia severity also showed a significant difference by grade level (*t*=-2.29, *p*<.05). Friendship quality varied significantly based on whether the participant was an only child (*t*=2.15, *p*<.05) and household registration (*t*=2.63, *p*<.01). Additionally, both friendship quality (*F*=4.67, *p*<.01) and family functioning (*F*=11.79, *p*<.001) showed significant differences based on family economic conditions.

**Table 3:** Distribution of research variables across demographic characteristics.

Demographic Variables	Peer Victimization	Friendship Quality	Family Functioning	Insomnia Severity
Gender				
Male	0.41 ± 0.58	3.34 ± 0.89	1.13 ± 0.69	1.02 ± 0.60
Female	0.42 ± 0.53	3.55 ± 0.80	0.97 ± 0.67	1.17 ± 0.68
*t*	-0.30	-2.83**	2.57*	-2.68**
Grade				
Junior high school year one	0.42 ± 0.59	3.45 ± 0.89	1.07 ± 0.70	1.03 ± 0.62
Junior high school year two	0.40 ± 0.52	3.46 ± 0.81	1.02 ± 0.67	1.16 ± 0.67
*t*	0.44	-0.04	0.78	-2.29*
Only Child				
Yes	0.38 ± 0.54	3.53 ± 0.84	1.08 ± 0.68	1.07 ± 0.62
No	0.45 ± 0.57	3.37 ± 0.85	1.00 ± 0.68	1.14 ± 0.67
*t*	-1.55	2.15*	1.45	-1.27
Household Registration				
Urban	0.38 ± 0.54	3.61 ± 0.83	1.09 ± 0.67	1.18 ± 0.68
Rural	0.43 ± 0.56	3.39 ± 0.85	1.02 ± 0.69	1.07 ± 0.64
*t*	-0.81	2.63**	1.08	1.73
Family Economic Status				
Poor	0.51 ± 0.60	3.06 ± 0.74	0.73 ± 0.53	1.19 ± 0.73
Average	0.46 ± 0.57	3.38 ± 0.82	0.94 ± 0.65	1.13 ± 0.65
Good	0.34 ± 0.52	3.59 ± 0.86	1.20 ± 0.70	1.05 ± 0.64
Wealthy	0.24 ± 0.42	3.81 ± 1.09	1.55 ± 0.47	1.18 ± 0.69
*F*	2.41	4.67**	11.79***	0.72

*p <.05, **p <.01, ***p <.001.

### 3.3 Correlation analysis

**Table 4** shows the correlations between variables. Peer victimization (*r*=.44, *p*<.001), physical victimization (*r*=.26, *p*<.001), and relational victimization (*r*=.46, *p*<.001) were significantly positively correlated with insomnia severity. Peer victimization (*r*=-.26, *p*<.001), physical victimization (*r*=-.18, *p*<.001), and relational victimization (*r*=-.26, *p*<.001) were significantly negatively correlated with family functioning. Family functioning was significantly negatively correlated with insomnia severity (*r*=-.31, *p*<.001). Friendship quality did not show significant correlations with peer victimization (*r*=-.06, *p*>.05) or insomnia severity (*r*=-.05, *p*>.05) but was significantly positively correlated with family functioning (*r*=.32, *p*<.001).

**Table 4:** Descriptive statistic and correlation matrix for variables.

Variable	*M*	*SD*	1	2	3	4	5	6	7	8	9	10	11	12
1.Peer Victimization	0.41	0.55	1											
2.Physical Victimization	0.37	0.59	.78***	1										
3.Relational Victimization	0.43	0.60	.97***	.61***	1									
4.Friendship Quality	3.46	0.85	-.06	-.04	-.06	1								
5.Companionship and Recreation	3.97	1.03	-.06	-.04	-.06	.85***	1							
6.Intimacy Exchange	3.67	1.14	-.04	-.06	-.03	.89***	.72***	1						
7.Validation and Caring	3.57	1.09	-.08	-.06	-.08	.87***	.70***	.76***	1					
8.Conflict Resolution Strategies	3.80	1.07	-.11*	-.10*	-.10*	.86***	.73***	.73***	.70***	1				
9.Help and Guidance	3.60	1.12	-.12**	-.09*	-.12**	.86***	.69***	.73***	.72***	.75***	1			
10.Conflict and Betrayal	3.87	1.00	-.16***	-.16***	-.14**	-.38***	-.17***	-.22***	-.18***	-.12**	-.14**	1		
11.Family Functioning	1.04	0.68	-.26***	-.18***	-.26***	.32***	.24***	.28***	.36***	.29***	.31***	.003	1	
12.Insomnia Severity	1.10	0.65	.44***	.26***	.46***	-.05	-.03	-.03	-.06	-.07	-.10*	-.07	-.31***	1

*p <.05, **p <.01, ***p <.001.

### 3.4 Hierarchical regression analysis

Hierarchical regression analysis was used to test the hypothesis. Taking insomnia severity as the outcome variable, gender, grade, only child, household registration and family economic conditions as control variables were first put into the first layer, then peer victimization and family functioning were put into the second layer, and finally the decentralized interaction terms between peer victimization and family functioning were put into the third layer. The same method was used to examine interaction between peer victimization and friendship quality. Finally, both family functioning and friendship quality were put into the model for further verification.

Hierarchical regression analysis results (see **Table 5**) indicated that peer victimization had a significant positive effect on insomnia severity *(β*=0.47, *t*=9.99, *p*<.001), supporting Hypothesis 1. The interaction term between peer victimization and friendship quality did not significantly predict insomnia severity (*β*=-0.03, *t*=-0.56, *p*>.05), so Hypothesis 2 was not supported. However, the interaction term between peer victimization and family functioning significantly predicted insomnia severity (*β*=-0.16, *t*=-2.58, *p*<.05), indicating that higher family functioning buffering the effect of peer victimization on insomnia severity, thus supporting Hypothesis 3.

**Table 5:** Moderation effect test.

Variable	Model 1		Model 2		Model 3		Model 4		Model 5		Model 6		Model 7	
	*β*	*t*	*β*	*t*	*β*	*t*	*β*	*t*	*β*	*t*	*β*	*t*	*β*	*t*
Gender	0.15	2.63^**^	0.12	2.34^*^	0.11	2.27*	0.16	3.00^**^	0.16	3.08^**^	0.11	2.22*	0.11	2.19^*^
Grade	0.13	2.27^*^	0.14	2.73^**^	0.13	2.69**	0.15	2.84^**^	0.15	2.87^**^	0.14	2.71**	0.13	2.69^**^
Only Child	0.06	1.01	0.02	0.32	0.03	0.54	0.02	0.34	0.02	0.36	0.02	0.35	0.03	0.57
Household Registration	-0.12	-1.87	-0.13	-2.46^*^	-0.12	-2.26*	-0.14	-2.43^*^	-0.14	-2.48^*^	-0.13	-2.40*	-0.12	-2.23^*^
Family Economic Status	-0.05	-1.15	0.04	1.01	0.05	1.10	0.01	0.13	0.01	0.22	0.04	0.96	0.05	1.09
Peer Victimization			0.47	10.02^***^	0.43	8.78^***^	0.51	11.26^***^	0.52	11.24^***^	0.47	9.99^***^	0.43	8.74^**^
Family Functioning			-0.20	-5.16^***^	-0.20	-5.21^***^					-0.21	-5.04^***^	-0.21	-5.10^**^
Friendship Quality							-0.04	-1.15	-0.03	-1.09	0.01	0.44	0.01	0.46
Peer Victimization × Family Functioning					-0.16	-2.58^*^							-0.15	-2.11^*^
Peer Victimization × Friendship Quality									-0.07	-1.41			-0.03	-0.56
*R^2^*	.04		.27		.28		.23		.24		.27		.28	
*ΔR^2^*	.04		.24		.01		.20		.003		.24		.0005	
*ΔF*	3.64^**^		80.46^***^		6.64^*^		64.54^***^		1.99		53.61^***^		0.31	
*F*	3.64^**^		26.42^***^		24.21^***^		21.70^***^		19.28^***^		23.10^***^		19.36^***^	

*p <.05, **p <.01, ***p <.001.

**Figure 1:**
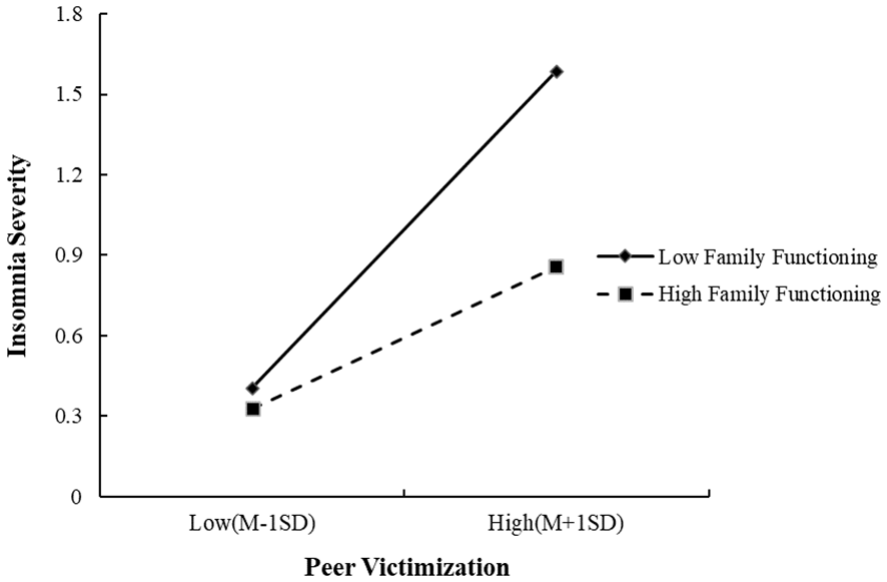
The moderating effect of family functioning on the relationship between peer victimization and insomnia severity.

To further validate the moderating effect, a moderation plot was created to show the relationship between peer victimization and insomnia severity at one standard deviation above and below the mean level of family functioning. As shown in **Figure 1**, the moderating effect of family functioning was significant with a 95% CI = [-0.29, -0.04], *β*=-0.16, *p*<.05. The impact of peer victimization on insomnia was greater when family functioning was lower (*β*=0.54, *t*=9.98, *p*<.001) compared to when it was higher (*β*=0.32, *t*=4.26, *p*<.001), demonstrating a significant negative moderating effect of family functioning and further supporting Hypothesis 3.

## 4 Discussion

This study focused on adolescents to explore the predictive role of peer victimization on insomnia severity and the interactive effects of peer victimization with family functioning and friendship quality. The goal was to identify factors that could moderate the negative impact of peer victimization on insomnia.

Our study found that 40.32% of adolescents experienced varying degrees of insomnia, which significantly affected their daytime functioning and quality of life. This finding aligned with Chung (32), while the students of this study were from non-graduating class, and students in graduating class may face higher levels of insomnia. They face higher academic pressures, often studying late into the night or worrying about their future (33). Insomnia severity was notably higher in females compared to males, which is related to hormonal changes in women after menstruation, greater stress levels and stress response (34).

Over half of the adolescents (64.03%) reported experiencing peer victimization, with no significant gender differences. Previous studies also found that the sex ratio was similar (35, 36), but the overall proportion of peer victimization was 25.7% (36), which was much lower than the result of this study. This discrepancy may be due to differences in the scales used to measure peer victimization and significant societal changes over the past decade, such as reduced emotional investment from parents in education, increased academic pressure in schools, and the rise of online bullying, which have all contributed to a higher prevalence of peer victimization.

Consistent with our hypothesis, both correlation and regression analyses revealed a significant positive impact of peer victimization on insomnia severity among adolescents. Previous research has also confirmed that adolescents who experience more peer victimization are likely to transition from having good sleep to poor sleep over time, with such changes potentially occurring earlier in males (37). The underlying mechanism involves peer victimization affecting brain function and behavior, particularly activating brain regions related to threat processing, reward, social pain, and emotions, such as the amygdala, the left parahippocampal gyrus, and the fusiform gyrus (38), and it can impair the hypothalamic-pituitary-adrenal (HPA) axis (through cortisol’s influence on the circadian rhythm) (39), which regulates alertness and sleep, potentially leading to insomnia, anxiety, depression, and behavioral issues. Educators can help adolescents to explore and strengthen reasonable beliefs and behaviors, improve cognitive, social and emotional skills through play and exercise interventions (40), or recruit peer educators to carry out relevant activities with peers in the classroom to develop empathy for victims of peer victimization and learn coping strategies to reduce peer victimization (41).

Regarding the moderating role of family functioning, our results supported the hypothesis. Specifically, the impact of peer victimization on insomnia severity was greater when family functioning was poor compared to when it was good. Extensive evidence suggests that the family environment and parenting styles significantly influence adolescent sleep. For example, adolescents from nuclear families have a lower risk of insomnia compared to those from single-parent or blended families (42), and family support is negatively correlated with insomnia (43). Good family functioning can provide children with a sense of security, enhances coping and emotional regulation through effective communication and support, which is associated with less insomnia. Additionally, research showed that adolescents’ sleep attitudes and patterns are similar to those of their parents (44), and according to social learning theory, the attitudes and behaviors of primary caregivers in the family are also potential factors. This suggests that improving parents’ parenting skills and sleep habits could enhance adolescents’ sleep quality. Conversely, a longitudinal study from early childhood to adolescence found that family functioning and marital conflict did not predict insomnia (45). This discrepancy may be due to differences in insomnia diagnostic criteria; the study used a strict DSM-IV definition of insomnia, whereas our study used the more lenient ISI scale.

Our study did not find a significant role of friendship quality in the relationship between peer victimization and insomnia, and several studies have also found no moderating effect of friendship quality. For instance, cross-sectional research has shown that public victimization among adolescents is significantly positively correlated with high levels of rule-breaking behavior, with peer social support providing no protective effect (46). Additionally, a longitudinal study tracking the effects of peer victimization on depressive symptoms in young adulthood also found no association with levels of friendship support (47). We hypothesize that friendship quality might not directly impact insomnia but is related to overall mental health (48). According to the stress-buffering model, adolescents who experience peer victimization through social support can reduce the negative impact of negative events and indirectly help reduce insomnia (49). However, among adolescent girls, high levels of intimacy in friendships might exacerbate maladaptive responses and distress caused by peer victimization (50). Another possible reason is that adolescents who suffer from peer victimization will be classified as unwelcome groups, shaking the original friendship system and becoming easy to be isolated (51). Further, they will seek out new friends with high prosocial levels or with different victimization status (52), to help mitigate the negative effects of peer victimization. Since our study is cross-sectional, it could not capture the impact of changing friendship quality over time, and some adolescents might consider their best friendships to exist in the virtual world.

Our study also has limitations. Firstly, the participants in this study were all from a single junior high school in Hubei province, and geographical specificity may limit the generalizability of findings. Secondly, as a cross-sectional study, it cannot clarify the causal relationships between peer victimization, family functioning, friendship quality, and insomnia severity, nor determine whether family functioning and friendship quality precede and contribute to peer victimization. Lastly, the self-report nature of the study might introduce social desirability bias, affecting the accuracy of the results. Future research should combine cross-sectional and longitudinal designs to explore gender effects more comprehensively, incorporate interviews with parents and teachers, and provide new insights into preventing and intervening in peer victimization.

## 5 Conclusion

In conclusion, this study found that family functioning, but not friendship quality, negatively moderated the relationship between peer victimization and insomnia. The results highlight that good family functioning can help adolescents reduce the negative impact of peer victimization on sleep. Educators and clinicians working with adolescents should encourage parents to enhance communication with their children and provide stable emotional support and assistance to help adolescents cope with negative events properly and reduce the occurrence of insomnia.

## Data Availability

The original contributions presented in the study are included in the article/supplementary material. Further inquiries can be directed to the corresponding authors.
